# Accuracy of an Affordable Smartphone-Based Teledermoscopy System for Color Measurements in Canine Skin

**DOI:** 10.3390/s20216234

**Published:** 2020-10-31

**Authors:** Blaž Cugmas, Eva Štruc

**Affiliations:** 1 Biophotonics Laboratory, Institute of Atomic Physics and Spectroscopy, University of Latvia, 19 Rainis Blvd., LV-1586 Rīga, Latvia; 2 Vetamplify SIA, Veterinary Services, 57/59–32 Krišjāņa Valdemāra Str., LV-1010 Rīga, Latvia; eva@vetamplify.com

**Keywords:** color calibration, color constancy, smartphone teledermoscopy, colorchecker, erythema index, canine skin, veterinary dermatology, CIELAB color space, color correction, color calibration target

## Abstract

Quality smartphone cameras and affordable dermatoscopes have enabled teledermoscopy to become a popular medical and veterinary tool for analyzing skin lesions such as melanoma and erythema. However, smartphones acquire images in an unknown RGB color space, which prevents a standardized colorimetric skin analysis. In this work, we supplemented a typical veterinary teledermoscopy system with a conventional color calibration procedure, and we studied two mid-priced smartphones in evaluating native and erythematous canine skin color. In a laboratory setting with the ColorChecker, the teledermoscopy system reached CIELAB-based color differences Δ*E* of 1.8–6.6 (CIE76) and 1.1–4.5 (CIE94). Intra- and inter-smartphone variability resulted in the color differences (CIE76) of 0.1, and 2.0–3.9, depending on the selected color range. Preliminary clinical measurements showed that canine skin is less red and yellow (lower a* and b* for Δ*E* of 10.7) than standard Caucasian human skin. Estimating the severity of skin erythema with an erythema index led to errors between 0.5–3%. After constructing a color calibration model for each smartphone, we expedited clinical measurements without losing colorimetric accuracy by introducing a simple image normalization on a white standard. To conclude, the calibrated teledermoscopy system is fast and accurate enough for various colorimetric applications in veterinary dermatology.

## 1. Introduction

Smartphones have become a popular tool for monitoring human health and well-being [1]. Recent advances in device display, sensors, battery, and connectivity have enabled stand-alone detection of cardiovascular parameters (heart or respiratory rate), sleep patterns, activity levels, cognitive functions, and changed health conditions in skin and eyes [1]. In veterinary medicine, smartphones were similarly applied for activity monitoring, electrocardiography, and endoscopy in various pet and farm animals [2,3,4]. By attaching adapters, holders, and additional mechanical, electrical, or optical modules, smartphones can also be suitable for various laboratory applications [5]. Colorimetric analyses of urine- or blood-related strips [6] and immunochromatographic assays [7] are among the most popular. Furthermore, smartphones have been exploited as analyzers of the enzyme-linked immunosorbent assay (ELISA) microplates, microfluidic system modules, and microscopes [8].

Smartphones have also contributed to developing a new subfield of dermatology – smartphone-based teledermoscopy [9,10]. Optical systems joining capable smartphone cameras and mobile dermatoscopes allow users to take and share quality images of their skin lesions, which could serve as preventive screening for cancer [9], especially where professional assistance is not available face to face. Compared to in-person dermatological services, smartphone-based teledermoscopy achieved the same sensitivity but lower specificity for detecting melanoma [11]. Furthermore, teledermoscopy can be complemented with image processing algorithms for automated skin lesion classification [12]. Rizvi and others even speculated that many regular consultations on skin lesions with the specialist could be replaced by the use of smartphone applications [13].

In human dermatology, diagnosing skin lesions usually relies on a dermatoscope, enabling detailed lesion observation according to the ABCD rules or 7-point checklist [14]. However, because melanomas in animals are less frequent and malignant, veterinarians typically employ dermatoscopes for studying other skin conditions such as alopecia (e.g., hair loss) [15]. Our research group recently used an affordable ($300) dermatoscope, the DermLite DL1 basic (3Gen, Inc., San Juan Capistrano, CA, USA) for the objective estimation of skin erythema severity in atopic dogs [16]. There was a high correlation (*r* = 0.82–0.85) and prediction ability (*r*^2^ = 0.72–0.73) between a dermatologist’s visual erythema estimates and an erythema index (EI), calculated directly from the image RGB values.

However, each smartphone acquires images in the camera-dependent RGB color space due to the sensor’s unique spectral response, preventing standardized colorimetric skin assessments. One option to overcome this limitation is to perform a transformation into a device-independent color space (e.g., CIELAB [17]) with the help of color calibration targets (CCT). The optical systems and transformation between color spaces naturally cannot guarantee perfect color accuracy. Therefore, an error between the measured and reference color appears called color difference (Δ*E*). The original, CIE76, Euclidian distance-based formula for Δ*E* estimation was updated twice (i.e., CIE94, CIEDE2000), but expanded versions are less often listed in the literature.

The CIELAB color space-based color difference Δ*E* can be transposed to the human eye capability to spot a difference between two colors. The just-noticeable difference (JND) usually has a Δ*E* estimated to be 2.3, but the detection threshold can be up to 5.0 [17]. Based on the popular CCT, such as the ColorChecker, various optical systems achieved similar colorimetric accuracies. The stand-alone use of popular cameras (e.g., SX200 IS, Canon, Tokyo, Japan; D3100, Nikon, Tokyo, Japan; or ST60, Samsung Electronics Co. Ltd., Suwon, Korea) produced color differences (CIE76) in the range of 3.7–5.9 [18,19]. An attached dermatoscope (Dermlite Pro) lowered the CIE76 Δ*E* to between 2.5 and 4.0 [20]. Furthermore, a camera (IDS UI-2240-C, IDS Imaging Development Systems GmbH, Obersulm, Germany) attached to the microscope resulted in a CIE 94 color distance of 2.2 [21]. 

The color constancy of smartphones (Samsung Galaxy S4 and S7, iPhone 5S) was slightly worse, with Δ*Es* respectively ranging between 3.5–8.4 [22], 7.4–10.2 (CIE76) [23], and 3.0–4.4 (CIEDE2000) [24]. Finally, the optical system with a smartphone and a miniature microscope ensured the CIE 94 color difference between 2.0 and 2.4 [8].

This work’s overarching goal is to set up a color calibration on a typical veterinary smartphone-based teledermoscopy system and study the colorimetric accuracy of two mid-priced smartphone cameras on canine skin. First, we transformed the acquired data from device-dependent (RGB) to device-independent (CIELAB, sRGB) color space. We continued with estimating colorimetric accuracy in a laboratory setting on the standard color calibration target (CCT), ColorChecker. We also made preliminary measurements of canine skin in terms of CIELAB color and erythema indices (sRGB). Additionally, we evaluated color variability for each (intra-) and between both smartphones (inter-smartphone accuracy). Finally, we confirmed that a simple image normalization on the white standard could expedite clinical measurements without decreasing colorimetric accuracy.

## 2. Materials and Methods

### 2.1. Optical System

**Figure 1:**
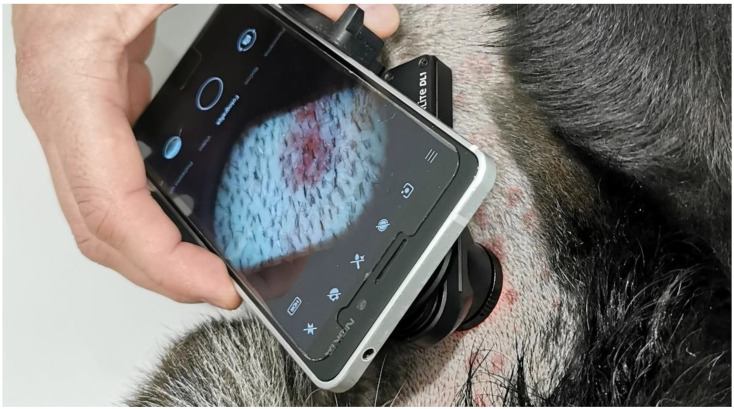
In this study, we applied a Smartphone-based teledermatoscopy system with a smartphone (one of two smartphones applied, SP1, Nokia 6 v.2017) and the DL1 basic dermatoscope for colorimetric measurements in canine skin.

We employed two mid-priced smartphones. The first (SP1) was a Nokia 6 smartphone (v.2017, HMD Global, Espoo, Finland) equipped with a 16 MP camera (f/2.0). JPEG images (90% quality, 4608 × 3456 pixels) were acquired in the program Open Camera (v1.47.3, Mark Harman, Cambridge, UK) with the following settings: photo mode (STD), white balance (manual, fluorescent), scene mode (steady photo), color effect (none), ISO (50), and focus (auto). The second smartphone (SP2) was a Samsung Galaxy A51 (Samsung Electronics Co. Ltd., Suwon, Korea) with a 48 MP camera (f/2.0). The images (90% quality, 4000 × 3000 pixels) were taken in the Open Camera program with the following settings: photo mode (STD), white balance (manual, cloudy), color effect (none), ISO (100), and focus (auto). To both smartphones, we attached DermLite DL1 basic a dermatoscope, an epiluminescence microscopy device with a 15 mm lens (Figure 1). The illumination consisted of four white LEDs with irradiance fluctuations being expected to be up to 0.9% [23]. Additionally, a polarizer reduced reflections from the skin surface. The camera was held 3.1 cm away from the skin, resulting in a circular 3.0 cm^2^ sampling area.

### 2.2. Laboratory Measurements

**Figure 2:**
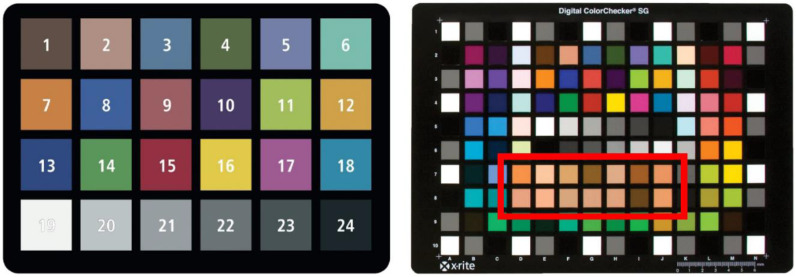
ColorChecker Classic (left, full-range CCT) and Digital SG (right, skin-range CCT). Skin patches used in our study are placed inside the red square.

Two ColorCheckers (Figure 2, X-rite, Grand Rapids, MI, USA) served as the reference color calibration targets (CCTs). For testing color calibration models on the whole color range, we exploited all patches on the Classic ColorChecker (full-range CCT). Only the white and skin patches (E5, D-J 7&8) on the Digital SG ColorChecker (skin-range CCT) were employed for skin color analysis.

**Figure 3:**
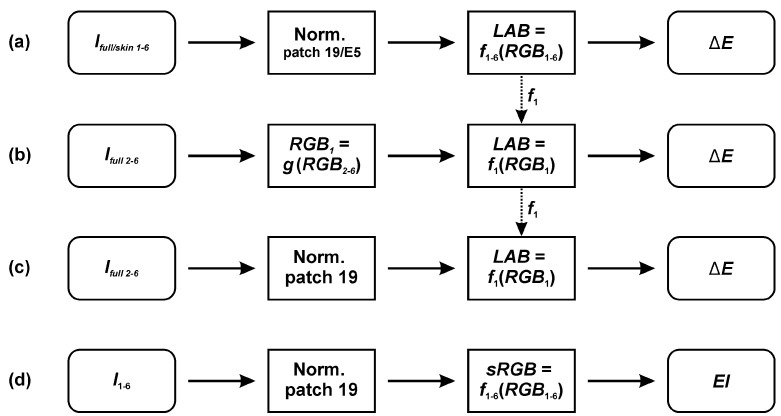
Flowcharts of different color calibration procedures. (**a**) Image sets (*I_full/skin_*_1–6_) of the selected CCT color patches were acquired and normalized against patches 19 (full-range CCT) or E5 (skin-range CCT). For each set independently, we built a transformation model between an unknown RGB and a standard CIELAB color space (*LAB* = *f*(*RGB*)). (**b**) Full normalization. RGB values of *I*_2–6_ were first transformed to the RGB color space of *I*_1_ and later to the CIELAB based on the known *f*_1_. (**c**) Single-point normalization. Normalized images (*I*_2–6_) with the brightest patch (No. 19) were directly transformed to the CIELAB color space based on the known *f*_1_. (**d**) Erythema Index (EI) was calculated after the colorimetric data was transformed to the standard RGB color space (sRGB).

Figure 3 displays flowcharts of different color calibration procedures, described below. First, we acquired six repeated images of each CCT (*I_full/skin_*_1–6_), thus having 12 individual image sets. For each set separately, the brightest white patch (19 for the full-range and E5 for the skin-range CCT) served for data normalization and setting a fixed camera exposure time, which did not change within image set. A median value of central 750 pixels in both dimensions served for the calculation of the image’s *RGB* values: *R* (red), *G* (green), and *B* (blue).

For each image set (Figure 3a), we then built a linear-regression model between an unknown device-dependent RGB and a device-independent CIELAB color space: *LAB* = *f_full/skin_*_1–6_(*RGB*). The mathematical model was based on the best performing polynomial from our previous study [23]:

(1) $$LAB=a_{0}+a_{1}R+a_{2}G+a_{3}B,\;\;\;\;\;\left\{L^{*},a^{*},b^{*}\right\}\in \;LAB,$$

where L* (lightness), a* (green-red), and b* (blue-yellow) are coordinates of the CIELAB color space, and *a*_0–3_ are regression coefficients, estimated with the function *fitlm* (Matlab, R2016a, MathWorks, Natick, MA, USA). The color difference (Δ*E*, CIE76) was calculated from fitting residuals as a Euclidean distance between estimated and true color [17]:

(2) $$\Delta E=\sqrt{{\left(\Delta L^{*}\right)}^{2}+{\left(\Delta a^{*}\right)}^{2}+{\left(\Delta b^{*}\right)}^{2}}.$$

CIE94′s Δ*E* was estimated with the openly available Matlab code [25].

Based on the full-range CCT, we additionally tested a few modified color calibration procedures, which could improve, deteriorate, simplify, or expedite clinical measurements:

(1) *Longer exposure time*. If the exposure time is not locked on the brightest CCT’s patch (e.g., patch 19), the camera sensor’s capacity limit can be reached, leading to saturation. We acquired additional six full-range CCT image sets (*S_full_*_1–6_) with the exposure time locked on the slightly gray patch (full-range CCT: patch 20, neutral 8 (.23 *), L* = 81.3) and a transformation (regression) model was built in the same manner as described before (Figure 3a). Finally, the retrieved color differences were calculated and compared to the calibration on the image sets *I_full/skin_*_1–6_.
(2) *Full normalization* (Figure 3b) was done across the whole lightness range based on the grayscale patches (No. 19–24). As guaranteeing a color constancy by acquiring all 24 patches for each clinical measurement could be burdensome and time-consuming, we expedited the color calibration procedure by introducing an initial transformation from the subsequent (*I_full_*_2–6_) to the first image set (*I_full_*_1_) RGB color space: *RGB*_1_ = *g(RGB*_2–6_*)*, where *g* was a quadratic polynomial. Six grayscale patch images served for the determination of function’s coefficients with the regression model fit. Finally, *RGB* values were converted to the CIELAB color space based on the known *f*_1_ (Figure 3a).
(3) *Single-point normalization* (Figure 3c) expedited a color calibration procedure since the normalization was implemented with only the brightest (No. 19) instead of the six grayscale patches. As before, the transformation to CIELAB color space was based on the known model *f*_1_ from the image set *I_full_*_1_.

### 2.3. Clinical Measurements

Clinical measurements were ethically approved by the Administration of the Republic of Slovenia for Food Safety, Veterinary Sector and Plant Protection under the number U3440-187/2020/7.

In addition to the image sets *I_full_*_1–6_, we acquired skin images of six dogs in the inguinal region. For each measurement site, we repeated the image acquisition on the same spot. The typical skin RGB values were calculated as the pixels mean from two manually selected small squares [26]. We transformed the RGB values into the CIELAB color space based on the known models *f*_1–6_ (Figure 3a). We additionally compared skin color estimates between repetitions (intra-smartphone variability) and both smartphones (inter-smartphone variability), according to Equation (2) above. Finally, *LAB* skin values were determined with a single-point normalization (Figure 3c) and compared to the original fits.

We calculated the erythema index (EI) for canine skin measurements and for the selected full-range CCT color patches (1–3, 5, 8, 9, and 19–24), which have physiological EI values. First, device-dependent RGB color data were transformed to the standard RGB color space (sRGB, Figure 3d) based on the reference values of grayscale patches (No. 19–24). Then, we calculated the erythema index *EI_BRG_*:

(3) $$EI_{BRG}=\frac{\sqrt{B\cdot R}}{G}.$$

We opted for an *EI_BRG_* since it was correlated the most with the canine erythema severity [26]. We additionally calculated EI with two additional (and faster) color calibration procedures (i.e., the full and single-point normalizations, Figure 3b,c). Finally, we studied agreements on EI estimation between both smartphones and against the full-range CCT reference values.

## 3. Results

**Figure 4:**
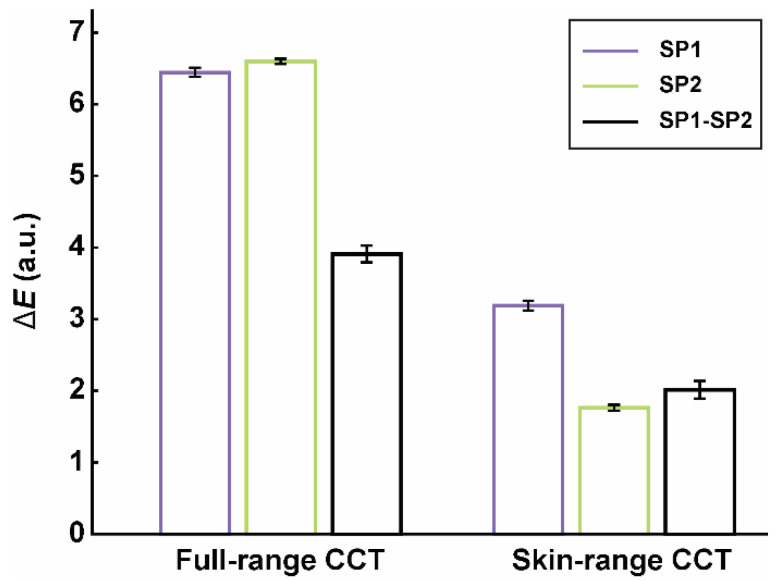
Mean CIE76 color differences (Δ*E*) with standard deviations as error bars for the full-range or skin-range CCT, obtained from the six image sets which were acquired by the first (SP1, Nokia) or second (SP2, Samsung) smartphone, respectively. SP1–SP2 marks the color differences between both smartphones’ estimates.

Laboratory measurements on the full-range CCT showed that the Nokia smartphone (SP1) achieved a slightly better color accuracy than the Samsung smartphone (SP2) since their average CIE76 color differences were 6.45 and 6.60, respectively (Figure 4, Table 1). The average variability in color differences for each smartphone (i.e., intra-smartphone variability) was up to CIE76 Δ*E* of 0.12 (CIE94 Δ*E* = 0.08). When we studied the differences in color estimates between both smartphones (i.e., inter-smartphone variability), CIE76 Δ*E* increased to 3.91 (CIE94 Δ*E* = 2.60). When only the skin color patches were involved in the color calibration, the color differences were significantly smaller (Figure 4, Table 1).

**Figure 5:**
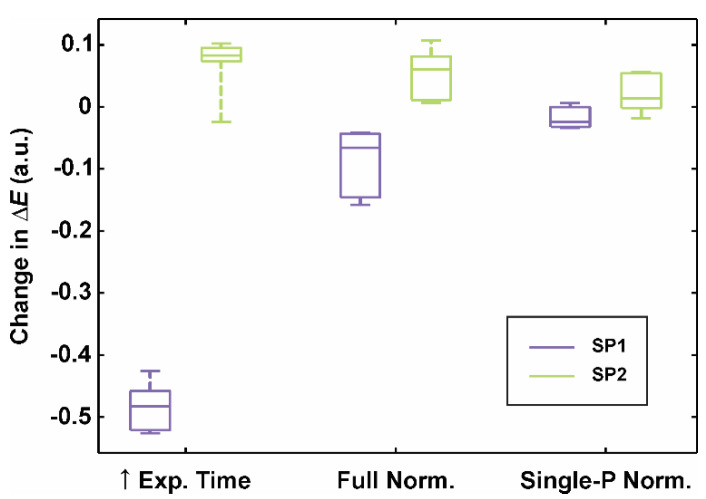
Changes in CIE76 color difference (Δ*E*) due to the modified color calibration procedures: increased exposure time reaching the sensor’ capacity limit (Exp. Time), full (Full Norm.), and single-point normalization (Single-P Norm.). A negative change marks the improvement in colorimetric accuracy. Boxplots: the central mark represents the median; the bottom and top box edges indicate the 25th and 75th percentiles, respectively. Whiskers extend to the most extreme data point.

Furthermore, the impact of all three modified color calibration procedures (i.e., a longer exposure time, or full-, or single-point normalizations) on the colorimetric accuracy was limited (Figure 5). On average, CIE76 Δ*E* fell for 0.48, when SP1 operated closer to its sensor’s capacity limit. On the other hand, P2′s CIE76 color difference increased by 0.07. Normalization procedure on the grayscale patches (i.e., full normalization) or the single white patch (single-point normalization) did not improve or deteriorate colorimetric accuracy significantly; the change in CIE76 color difference (Δ*E*) was between −0.16 and 0.11.

**Figure 6:**
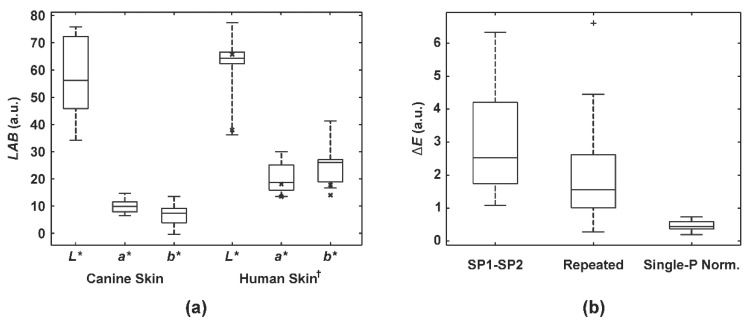
Clinical measurement of canine skin color. (**a**) Canine and human (full-range CCT: bold x-es, skin-range CCT: boxplots) skin color in the CIELAB color space. (**b**) Color differences (Δ*E*) in skin color between both smartphones’ estimates (SP1–SP2), between the original and repeated measurement (Repeated), and when a single-point normalization was applied (Single-P Norm.). Boxplots: the central mark represents the median; the bottom and top box edges indicate the 25th and 75th percentiles, respectively. Whiskers extend to the most extreme data point. The outlier is marked as a plus (+). († retrieved from the CCT specifications).

We performed preliminary clinical measurements on the skin of six dogs to determine and compare the *LAB* values to those of human skin based on the specifications from the full- and skin-range CCT (Figure 6a). Both, human and canine skin exhibited a similar lightness range, but the latter appeared to be less red and yellow (i.e., lower a* and b* values). Furthermore, the skin color estimations between both smartphones resulted in the mean CIE76 color difference of 2.97 (i.e., inter-smartphone variability, Figure 6b). The mean Δ*E* between the original and repeated color estimates of the single SP1 smartphone (i.e., intra-smartphone variability) was 2.18. Finally, when we studied the simplest color calibration of a single-point normalization on the brightest color patch (Figure 3c), the additional change in color difference was around 0.47.

**Figure 7:**
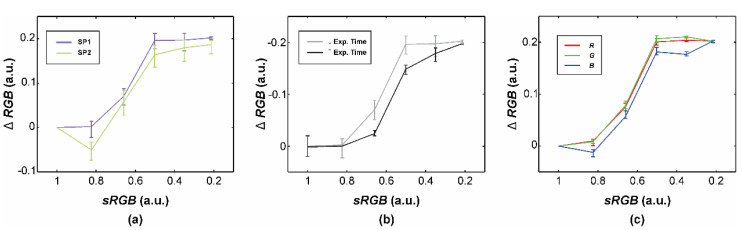
Absolute differences (Δ*RGB*) between the acquired and normalized referential *RGB* values (*sRGB*) comparing (**a**) both smartphones (SP1, SP2), (**b**) shorter (full-range CCT’s patch 19) and longer (patch 20) exposure times, and (**c**) separate SP1′s RGB channels. Units a.u. are retrieved from the normalization against the white standard (i.e., patches 19 or 20).

We additionally discovered significant absolute differences between the acquired and referential *RGB* values studying the grayscale patches on the full-range CCT (Figure 7). 

The first smartphone (SP1) generally produced smaller *RGB* differences on the bright patch (i.e., normalized referential *RGB* values of around 0.8), but the SP2-based system performed better with darker images (Figure 7a). However, both smartphones’ sensors responses were completely depressed while acquiring the black patch with a normalized referential *RGB* value (*sRGB*) of around 0.2 a.u. Furthermore, prolonging the exposure time by the image normalization on the gray patch 20 (instead of the white patch 19) significantly affected the sensor’s response with the increased *RGB* values up to 0.05 in the referential *RGB* range between 0.50 and 0.66 a.u. (Figure 7b). Although the differences to the standard grayscale CCT patches were smaller, the absolute differences remained significant, that is up to 0.20. Finally, we found that even individual RGB sensor elements have different spectral responses with channel-specific *RGB* differences of up to 0.04 a.u. (Figure 7c).

**Figure 8:**
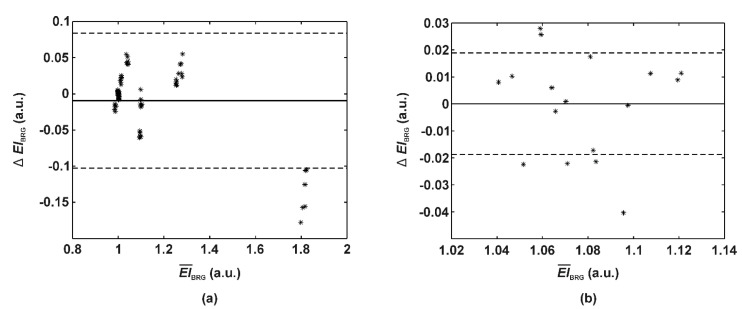
Bland-Altman plots on erythema index agreement between both smartphones. *EI_BRG_* is based on (**a**) the full-range CCT patches (No: 1–3, 5, 8, 9, and 19–24) or (**b**) canine skin. Mean difference and 95% limits of agreement (±1.96 STD) are marked as full and dotted lines, respectively.

Finally, we studied differences in erythema estimates (*EI_BRG_*) with various color calibration procedures (Table 2). The transformation to the standard RGB color space (sRGB) significantly improved the *EI_BRG_* agreement since absolute differences in erythema estimates dropped to around 0.04 compared to the calculations in the device-dependent RGB color space (0.29–0.60). We also found that the full normalization on six CCT patches did not outperform the shorter and simpler normalization with a single white patch. Repeated measurements on the CCT resulted in the *EI_BRG_* differences (i.e., intra-smartphone variability) of 0.005 and 0.003 for SP1 and SP2, respectively. Clinical skin measurements exhibited an increased intra-smartphone variability of 0.008 ± 0.007 and 0.011 ± 0.011 for SP1 and SP2, respectively. Single differences in *EI_BRG_* of the selected CCT patches and canine skin between both smartphones are shown in Figure 8.

## 4. Discussion

In this work, we characterized the color calibration using a typical veterinary smartphone-based teledermoscopy system. We studied its colorimetric accuracy in both laboratory (CIELAB color space with the color calibration target ColorChecker) and clinical settings (RGB-based erythema index of canine skin). We discovered that the calibrated teledermoscopy system is accurate enough since the CIELAB-based color differences (Δ*E*) were up to 6.6. Furthermore, estimating the severity of skin erythema with an erythema index led to errors lower than 3%. Finally, we showed that we could achieve comparably accurate colorimetrical measurements with a simple image normalization on the white surface without the need for time-consuming, full-color calibration.

In the laboratory settings on the full-range CCT, the proposed teledermoscopic setup with a smartphone and a dermatoscope achieved a decent color constancy with the average CIE76 color difference between 6 in 7 (Figure 4). Based on a polynomial of degree one with only four estimated parameters, a robust transformation model resulted in the color differences in the range of existing studies (Δ*E* between 2.5 and 10.2 [8,18,19,20,21,22,23]). However, some of the studies (e.g., Zhang et al. [8]) employed the quadratic polynomial with ten parameters included. When the same polynomial was applied to our measurements, the CIE76 Δ*E* decreased to 3.8 and 0.9 for the full- and skin-range CCT, respectively. Nevertheless, we do not recommend using a complex polynomial with a small number of measurements (in our case: 14–24) since it can easily lead to overfitting. Furthermore, we mostly listed the CIE76 color difference, which seemed to have an increased value in the used color range compared to the CIE94 and CIEDE2000 standards. For example, Δ*E* decreased to 3.9 (CIE94) or 3.8 (CIEDE2000) when the updated equations for color difference were applied. Furthermore, adding a complex polynomial to the transformation model resulted in the color difference (CIE94) of 2.3, which corresponds to the lower limit of the reported Δ*E* range [8].

Wang and Zhang [18] showed that utilizing CCT with a narrower color range halved the color differences (e.g., from 10.4 to 5.4). We observed a similar phenomenon since the transformation models on the skin-range CCT had a significantly lower Δ*E* (Figure 4, Δ*E* of 2–3). We also discovered comparably low color differences between the color estimates from both smartphones. Since the retrieved Δ*E* fell within the range of the human eye’s capability to spot a color difference (i.e., just noticeable difference, JND), our results point towards the adequacy of smartphones for dermatological use.

Selecting a longer exposure time (Figure 5) based on the gray patch (No. 20) increased the image’s RGB values. On the one hand, this improved the contrast of dark images, which would otherwise have similar, depressed *RGB* values. For example, longer exposure times decreased the percentage of low-intensity color patches (normalized *RGB* < 0.01) from 8.9% to 8.3% and from 15.0% to 13.1% for SP1 and SP2, respectively. On the other hand, prolonged exposures can lead the camera sensor to be close to its saturation charge level. The image pixels of the brightest patch (No. 19) held almost maximal *RGB* values, i.e., 0.92 and 0.96 for SP1 and SP2, respectively. Additionally, as shown in our previous study [23], brighter images with higher contrast can sometimes contribute to a better color constancy (e.g., with the first smartphone SP1). However, the effect of a longer exposure time can also have a negligible or even adverse impact, probably due to image saturation. Simultaneously, full range or single-point normalizations (Figure 3b,c) were more straightforward color calibration procedures. Both approaches sped up the clinical measurements without affecting the color constancy of both smartphones (Figure 5). Therefore, a single-point normalization is the most clinically appropriate since it is the fastest and the same accurate as full normalization.

We showed similarities between the lightness of human and canine skin in Figure 6a. However, it seems that canine skin is less red and yellow. Its average CIELAB’s a* and b* were 10.0 and 6.9, compared to 15.8 and 15.9 (full-range CCT), 20.2 and 25.3 (skin-range CCT) of human skin, resulting in color differences (CIE76 Δ*E*) of 10.7 (canine vs. human full-range CCT skin) and 10.3 (human full- vs. skin-range CCT skin). One could object that the color difference between our estimates and the full-range CCT skin specifications appeared due to the average smartphone error (Δ*E* = 6.5, Figure 4). However, our models (*f_full_*_1–6_) overestimated both skin patches (No. 1, 2) of the full-range CCT for Δ*E* of 6.0 (mean estimated and referential a* and b* values were 18.8 & 21.1, and 15.8 & 15.9, respectively). Therefore, misestimates due to the smartphone and model errors would compensate in the worst-case scenario, keeping the color difference between canine and human skin of around 10. On the other hand, the color differences between full- and skin-range CCT are expected since the semi-gloss finish of the skin-range CCT causes overestimates in a* and b* for Δ*E* of around 7 [27]. We should note that these conclusions are based on the standard human skin colors of the ColorChecker. If the normal canine skin color range was estimated, in vivo human skin measurements would contribute to the more real color comparison. However, these data will not improve the proposed optical system’s accuracy study since the actual colors (i.e., gold standard) would remain unknown.

Comparing skin color estimates between smartphones (Figure 6b) resulted in the Δ*E* of 3.0, similar to the results on the full-range CCT (Figure 4). However, we can attribute a significant portion of this color disagreement to the variability in clinical measurements (Δ*E* = 2.2). Since sampling the representative canine skin was done manually, it was a challenge to guarantee entirely repeatable skin area selection. Furthermore, a single-point normalization proved, again, to be sufficient for color calibration after the basic color transformation model for each smartphone was made.

This work and many other studies relied on the colorimetric parameters retrieved after the transformation between RGB and CIELAB color spaces, which guaranteed a certain level of colorimetric standardization. However, when a less experienced reader encounters parameters characterized directly in the device-dependent RGB color space, he or she could assume that these results are standardized and comparable between smartphones as well. As we showed in Figure 7 and Table 2, performing only an image normalization with a single reference point resulted in a significant disagreement (up to 60%) between both smartphones and the reference. We can attribute these misestimates in EI to two basic reasons. First, smartphone cameras (and their separate RGB channels) have individual spectral responses with different sensitivities to detect a certain lightness level (Figure 7a,c). For example, the first smartphone images had significantly lower absolute *RGB* values compared to the second smartphone. Secondly, the smartphone camera response is not linear; therefore, setting a specific exposure time affected the acquired data in the skin lightness range (Figure 7b). Therefore, we needed to construct a transformation to the device-independent standard RGB (sRGB) color space before any RGB-based parameter calculations (Figure 3d).

In our previous study [16], we estimated the *EI_RGB_* range of canine skin between 1.0 (healthy skin) and 2.0 (severely erythematous skin). After the transformation to sRGB, our teledermatoscopic setup achieved intra-smartphone EI variability up to 0.01, which corresponds to a 0.5–1.0% margin of error. Additionally, disagreements in the erythema index between both smartphones were, on average, around 0.03, matching an error between 1.5–3%. These results are superior to some of the other smartphone-based optical systems that assess skin chromophores such as hemoglobin [28], which reported disagreements between smartphones exceeding 25%.

Finally, we shall point out that only six dogs were enrolled in this study. However, it seems that 17 clinical (Figure 8b), in addition to the numerous laboratory measurements are sufficient for confirming the colorimetric reliability of the proposed optical system. Moreover, the results indicated that the measurement site (re-)selection and image sampling are significant factors for the color disagreement (Figure 6b). Therefore, the measuring and sampling procedures should be better specified before any comprehensive clinical study.

## 5. Conclusions

Although smartphone cameras acquire images in an unknown RGB color space, a simple color space transformation ensured accurate colorimetric measurements with color differences Δ*E* (CIE76) of 1.8–6.6 (CIE94 Δ*E* = 1.1–4.5). Since the results are within the range of JND (i.e., the human eye’s capability to spot a color difference), the proposed teledermoscopic optical setup with a smartphone and dermatoscope has the potential to replace the visual skin color assessment. We assume that we could achieve even better color constancy with the help of canine skin-tailored color patches. However, native canine skin color should be determined first. Besides the erythema index, we could study additional colorimetric parameters for erythema estimation (e.g., a* of the CIELAB color space). To conclude, the proposed affordable teledermatoscopy system has the capability to be employed in colorimetric studies in veterinary dermatology.

## Figures and Tables

**Table 1:** The color difference (Δ*E*) means and the whole ranges for individual patches (in brackets) on the full- and skin-range CCTs for the first (SP1) and second (SP2) smartphone and between both smartphones (SP1-SP2, i.e., inter-smartphone variability).

Color Calibration	SP1	SP2	SP1–SP2
Full-range CCT (CIE76)	6.45 (2.52–13.19)	6.60 (1.66–12.80)	3.91 (0.36–9.30)
Full-range CCT (CIE94)	3.96 (1.51–9.25)	4.46 (1.02–9.75)	2.60 (0.36–7.28)
Skin-range CCT (CIE76)	3.19 (0.93-9.04)	1.76 (0.47–3.65)	2.01 (0.34–7.18)
Skin-range CCT (CIE94)	1.74 (0.40–3.64)	1.16 (0.25–3.10)	1.05 (0.13–3.08)

**Table 2:** Absolute differences in *EI_BRG_* between CCT’s referential data and both smartphones’ estimates (SP1, SP2) with different EI color calibration procedures. 1: EI was calculated directly from the device-dependent SP1′s and SP2′s RGB color spaces, 2: Colorimetric data was transformed to the standard RGB color space (sRGB) by the full normalization on six grayscale patches, 3 and 4: Colorimetric data was transformed to the standard RGB (sRGB) color space by the single-point normalization on the brightest white CCT patch (No. 19).

Color Calibration	CCT–SP1	CCT–SP2	SP1–SP2
1. Without (RGB) ^L^	0.40 ± 0.93	0.60 ± 1.16	0.29 ± 0.40
2. Full Norm. (sRGB) ^L^	0.04 ± 0.06	0.04 ± 0.09	0.03 ± 0.04
3. Single-P Norm. (sRGB) ^L^	0.04 ± 0.06	0.04 ± 0.10	0.03 ± 0.04
4. Single-P Norm. (sRGB) ^C^	/	/	0.02 ± 0.01

^L^ Laboratory settings with the full-range CCT. ^C^ Clinical skin measurements.
